# Two mutations in NS2B are responsible for attenuation of the yellow fever virus (YFV) vaccine strain 17D

**DOI:** 10.1371/journal.ppat.1013373

**Published:** 2025-07-31

**Authors:** Xueer Qiu, Adam L. Bailey

**Affiliations:** 1 Department of Pathology and Laboratory Medicine, School of Medicine and Public Health, University of Wisconsin-Madison, Madison, Wisconsin, United States of America; 2 Microbiology Doctoral Training Program, University of Wisconsin-Madison, Madison, Wisconsin, United States of America; Fundación Instituto Leloir-CONICET, ARGENTINA

## Abstract

Vaccines have done more to improve the health of humankind over the past century than almost any other technology. Among vaccines, the live-attenuated yellow fever (YF) vaccine (17D) is highly effective, providing long-lasting immunity against yellow fever virus (YFV) infection with a single dose. Developed in the 1930s through extensive serial passage of the virulent YFV-Asibi strain through mouse and chicken embryonic tissue, 17D acquired several mutations that render it attenuated in humans and non-human primates. Over the past century, 17D has become a widely studied immunogen and has also been developed into a vaccine platform for other pathogens. Despite this, most studies of 17D have focused exclusively on the host, without clearly defining the virus-intrinsic features of attenuation. Consequently, the genetic determinants of 17D attenuation remain unknown and are assumed to be multigenic. Here, we leverage the hamster host, which recapitulates many important features of human YF disease, to understand the genetic basis of 17D attenuation. We developed a YFV reverse genetics system and generated hamster-adapted Asibi/17D chimeric viruses, discovering that viruses containing 17D-derived mutations in the viral gene NS2B were significantly attenuated in the hamster. Further analysis revealed that the two non-synonymous mutations in NS2B that distinguish 17D from Asibi, I37L and I109L, act cooperatively to mediate attenuation, with both mutations required to fully prevent key features of YF disease including liver injury and coagulopathy. These findings establish NS2B as an important and unexpected determinant of YFV-17D attenuation in vivo. In addition to the implications of these findings for improving the efficacy and safety of the 17D vaccine platform, this discovery also provides a new direction for understanding more generalized principles and mechanisms of durable vaccine-induced immunity.

## Author summary

Vaccines against deadly infectious diseases are a foundational technology of modern human civilization. Among vaccines, the live-attenuated yellow fever vaccine is one of the most effective. This vaccine strain (known as 17D) acquired 23 unique mutations during its attenuation process in the 1930s, yet the specific mutations that resulted in attenuation have never been pin-pointed. In other words, how do these mutations “code for” virulence versus attenuation? Here, we used recombinant DNA technology to create viruses that contain fragments from 17D and its virulent parent, Asibi. We then used these chimeric viruses to map the genetic determinants of 17D attenuation, identifying two subtle 17D-derived mutations in the viral NS2B gene that significantly impacted Asibi virulence in the hamster. This finding has direct implications for understanding yellow fever pathogenesis and yellow fever vaccine safety. More broadly, this discovery provides a new direction for understanding more generalized principles and mechanisms of durable vaccine-induced immunity.

## Introduction

Yellow fever virus (YFV), the prototype flavivirus, remains a significant public health threat in tropical and subtropical regions of Africa and South America. Human YFV infection is often severe and characterized by fever, jaundice, hemorrhage, and multi-organ failure––a pattern of disease historically referred to as viscerotropic yellow fever (YF). Despite the availability of an effective vaccine, spillover of YFV from wild nonhuman primate reservoirs causes frequent outbreaks of YF in humans, with an estimated 84,000–170,000 severe infections and 29,000–60,000 deaths annually in Africa alone [1,2].

Aside from its direct impact on combating YF, the live-attenuated YFV vaccine––commonly known as “17D”–– has played a defining role in the fields of vaccinology and immunology. Developed by Max Theiler and colleagues in the 1930s, YFV-17D was the product of a massive multi-year experiment that aimed to attenuate YFV by serial passage of the virulent YFV-Asibi strain through multiple combinations of mouse, chicken, and denervated chicken embryonic tissues. To ensure that infectious YFV remained present in the culture with each passage, supernatant was intracranially inoculated into mice––a route that bypasses the intrinsic resistance of the murine host to YFV infection and facilitates lethal neurotropic infection. At passage 176, the “17D” lineage was found to cause sub-lethal infection in mice; additional infection studies in macaque monkeys confirmed that 17D was indeed attenuated and also conferred robust immunity against challenge with the original highly virulent Asibi strain. After several more passages, the descendants of 17D, known as substrains 17D-204, -213 and -DD (i.e., passage 204, 239 and 287–288, respectively) became the live-attenuated vaccines used in humans since 1937 [3,4].

One dose of YFV-17D confers life-long immunity in ~90% of individuals, making it an attractive platform for the development of 17D-based vaccines against other flaviviruses [5–7]. Indeed, some of these––such as the chimeric 17D/Japanese encephalitis virus (JEV) vaccine (Imojev) that contains the JEV premembrane and envelope on a 17D “backbone”––have had moderate success. However, others––in particular, the 17D chimeric West Nile (PreveNile) and Dengue (Dengvaxia) vaccines––have been recalled or discontinued due to substantial safety concerns [8–10]. These failures are complicated by the fact that 17D itself has a well-documented history of rare but serious adverse events that exhibit many features of infection with virulent YFV [11,12].

Sequencing of the 17D-204, -213, and -DD substrains and comparison to the parental Asibi strain identified 48 mutations across the genome of 10,862 nucleotides that are common to all three major 17D lineages, 23 of which alter the encoded amino acid (Table 1) [6,13,14]. Importantly however, the genetic determinants of 17D attenuation remain poorly defined and are believed to be multifactorial [5]. Although 17D and Asibi have been shown to exhibit several in vitro biological differences including variations in viral entry, genetic diversity, and interferon induction, these findings have not translated into reliable predictors of attenuation in vivo [15–18]. In particular, studies comparing their replication kinetics have yielded cell type-dependent differences: 17D replicates more efficiently in some cells (e.g., HepG2, monocyte-derived dendritic cells) while others favor Asibi (e.g., Vero, PH5CH8 hepatocytes, primary Kupffer cells, endothelial cells) [15,19–23]. Thus, in vitro growth appears to be highly context-dependent and cannot serve as a reliable proxy for YFV virulence or attenuation in humans. To date, only the macaque monkey––which develops a “viscerotropic” pattern of disease akin to YF in humans––has historically been used to unequivocally distinguish attenuated from virulent YFV. Thus, there remains an outstanding need for the development of host systems that can differentiate 17D from Asibi in a moderate-throughput yet clinically meaningful manner.

**Table 1:** Genetic differences between YFV-Asibi isolates and YFV-17D substrains.

Gene	Codon in Gene	Codon Position	Asibi				17D			
			(AY640589)	FC (MT956630)	LSHTM (MT956629)	Yale (MT956628)	204 (KF769015)	204 (MN708488)	213 (YFU17067)	DD (YFU17066)
prM	125	246	L	L	L	L	F	F	F	F
prM	156	277	V	A	V	V	V	V	V	V
E	27	312	Q	H	Q	Q	Q	Q	Q	Q
E	52	337	G	G	G	G	R	R	R	R
E	56	341	A	A	A	A	V	V	V	A
E	153	438	N	N	N	N	T	N	T	N
E	155	440	D	D	A	D	D	D	D	S
E	170	455	A	A	A	A	V	V	V	V
E	173	458	T	T	T	T	I	I	I	I
E	199	484	E	G	E	E	E	E	E	E
E	200	485	K	K	K	K	T	T	T	T
E	299	584	M	M	M	M	I	I	I	I
E	305	590	S	S	S	S	F	F	F	F
E	325	610	P	P	P	P	S	S	S	P
E	331	616	K	K	R	K	R	R	R	R
E	380	665	T	T	T	T	R	R	R	R
E	407	692	A	A	A	A	V	V	V	V
E	416	701	A	A	A	A	T	T	T	V
NS1	79	857	L	L	L	L	F	F	F	F
NS1	307	1085	I	I	I	I	V	V	V	V
NS2A	31	1161	L	L	L	L	L	L	L	L
NS2A	105	1235	T	T	A	T	T	T	T	T
NS2A	118	1248	M	M	M	M	V	V	V	V
NS2A	167	1297	T	T	T	T	A	A	A	A
NS2A	169	1299	L	L	L	L	F	F	F	L
NS2A	172	1302	T	T	T	T	A	A	A	A
NS2A	183	1313	S	S	S	S	F	F	F	F
NS2B	37	1391	I	I	I	I	L	L	L	L
NS2B	109	1463	I	I	I	I	L	L	L	L
NS3	182	1666	Q	Q	Q	Q	Q	Q	Q	R
NS3	195	1679	I	I	I	I	V	V	V	I
NS3	485	1969	D	D	D	D	N	N	N	N
NS4A	146	2253	V	V	V	V	A	A	A	A
NS4B	95	2351	I	I	I	I	M	M	M	M
NS4B	98	2354	V	V	I	V	V	V	V	V
NS4B	204	2460	L	L	L	L	L	L	S	L
NS4B	232	2488	Y	Y	Y	Y	H	H	H	H
NS5	22	2528	Q	Q	Q	Q	R	R	R	Q
NS5	391	2897	N	N	N	N	N	N	N	S
NS5	836	3342	E	E	E	E	K	K	K	K
NS5	901	3407	P	P	P	P	L	L	L	L
3’UTR	−	10367*	T	T	T	T	C	C	C	C
3’UTR	−	10418*	T	T	T	T	C	C	C	C
3’UTR	−	10550*	T	T	T	T	C	C	C	T
3’UTR	−	10800*	G	A	G	A	A	A	A	A
3’UTR	−	10847	A				C	C	C	C

Asibi sequences: Asibi, AY640589; Asibi-FC, MT956630; Asibi-LSHTM, MT966629; Asibi-Yale, MT956628. 17D sequences: 17D-204, KF769015 and MN708488; 17D-213, YFU17067; 17DD, YFU17066.) AY640589 (Asibi) and MN708488 (17D-204) were used as reference genomes for all chimeric constructs and heatmap comparisons. Black denotes 23 non-synonymous mutations and 3 single nucleotide polymorphisms (SNPs) in the 3′ untranslated region (3′UTR) that distinguish all Asibi isolates from the vaccine strains. Blue denotes positions with mutations that are not unique among Asibi or 17D strains (and were excluded from this study). Grey-shaded cells represent regions with low sequencing coverage. CDS: coding sequence. Asterix indicate nucleotide position instead of codon.

In 2003 a hamster-adapted Asibi strain (HA-Asibi) was developed by serial passage of liver homogenate from an Asibi-infected hamster until virulence was attained at passage 7 [24]. HA-Asibi infection in hamsters recapitulates many important features of human YF including high-titer viremia, coagulopathy, and severe liver damage. Sequencing of HA-Asibi revealed 7 mutations relative to the parental Asibi strain, but genotype/phenotype mapping studies found that the mutation encoding a D155A change in the envelope (E) glycoprotein was responsible for the majority of HA-Asibi mediated disease in the hamster [25].

Here, we created a reverse genetics system for YFV using the circular polymerase extension reaction (CPER) method [26–28], enabling rapid creation of chimeric Asibi/17D viruses ± hamster-adapting mutations. Using viremia and weight loss in the hamster as primary readouts, we iteratively mapped the determinants of 17D attenuation, ultimately attributing attenuation to the combined effect of two isoleucine→leucine mutations in non-structural protein 2B (NS2B): I37L and I109L.

## Results

### Hamsters develop viscerotropic YF disease when infected with (hamster-adapted, HA)-Asibi but not 17D

**Fig 1:**
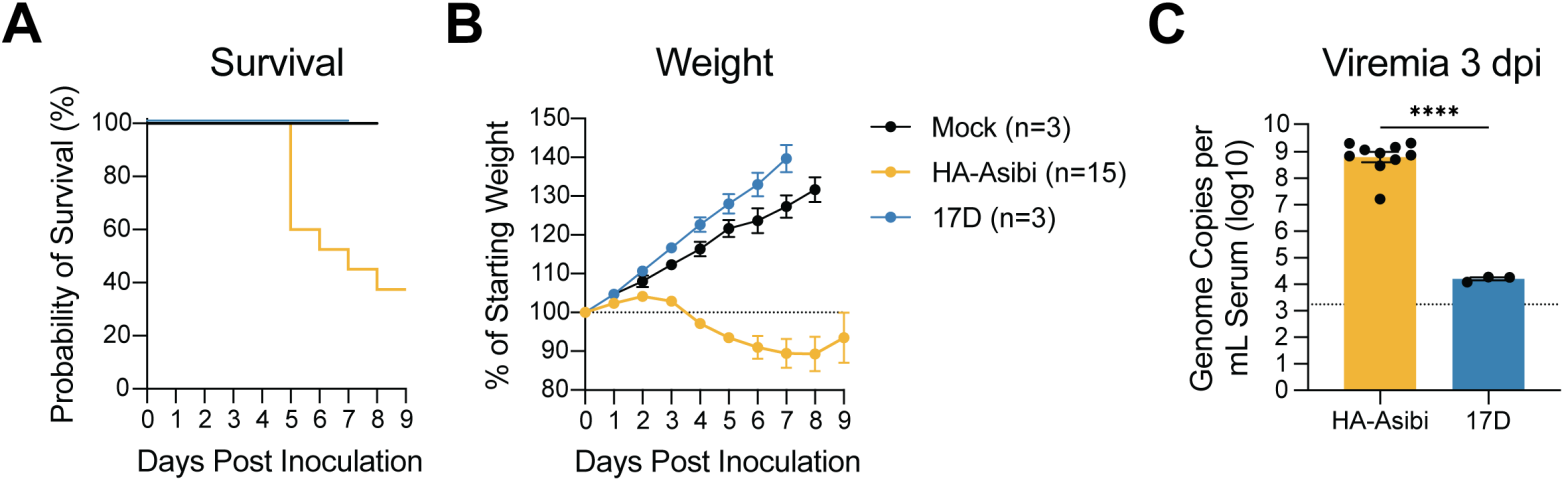
HA-Asibi but not 17D causes disease in hamsters. Hamsters were infected by intraperitoneal injection with 1 × 10^5^ focus-forming units (FFU) of HA-Asibi (yellow, n = 15), 17D (blue, n = 3), or mock (black, n = 3). Data represents a composite of 6 independent experiments. **(A)** Survival analysis, (B) weight change as a percentage of weight at the time of inoculation, and (C) viremia at 3 days post inoculation (dpi). Error bars show the mean ± standard error of mean (SEM). In C, the dashed line shows the limit of detection; statistical significance determined via unpaired two-tailed t test with Welch’s correction for unequal variance (****: p < 0.0001).

As established previously, Syrian golden hamsters infected with HA-Asibi developed disease characterized by high-titer viremia, weight loss, and death [29] (Fig 1A–1C). To determine whether 17D was attenuated in the hamster host, we inoculated hamsters with 17D-204 (subsequently referred to as “17D”). In comparison to HA-Asibi infected hamsters, 17D-infected hamsters did not develop disease and exhibited continuous weight gain despite detectable levels of viremia. (Fig 1A–1C). Given the divergent disease phenotypes between HA-Asibi and 17D, we hypothesized that the hamster could be a useful host for further investigating the determinants of 17D attenuation.

### CPER-generated recombinant YFVs behave like biological isolates

**Fig 2:**
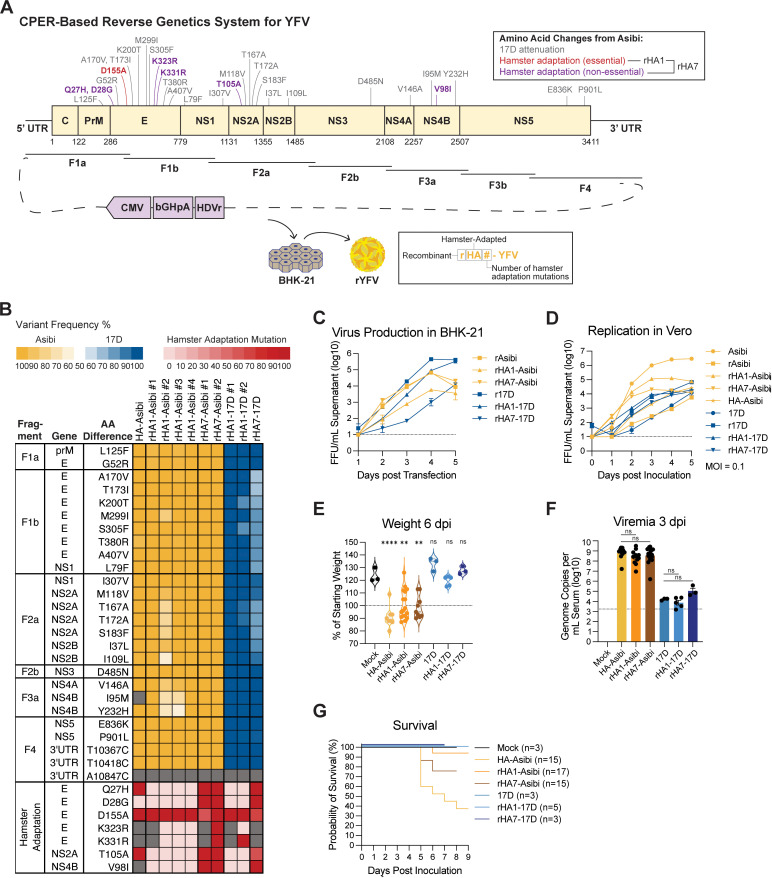
Recombinant HA-YFVs generated via CPER recapitulate disease phenotypes observed with biological isolates. **(A)** Schematic of the YFV genome showing amino acid differences in the polyprotein (yellow rectangle) between Asibi and 17D-204 (gray) along with mutations acquired during hamster adaptation of Asibi (purple with E-D155A shown in red). Overlapping DNA fragments amplified from YFV cDNA-containing plasmids are shown below, assembled in a circular polymerase extension reaction (CPER) with a linker sequence containing the CMV promoter and enhancer, bovine growth hormone polyadenylation (bGHpA) signal, and the self-cleaving hepatitis delta virus ribozyme (HDVr). The resulting circular CPER product was transfected into BHK-21 cells to rescue infectious recombinant YFV. The nomenclature for recombinant hamster-adapted YFV variants is shown in the box. **(B)** Heatmap showing the frequency of Asibi/17D mutations across biological and recombinant HA-YFVs. Viral genomes with an Asibi backbone were mapped to the Asibi reference (AY640589) and those with a 17D backbone were mapped to the 17D-204 reference (MN708488). Columns represent independently rescued virus stocks, and rows indicate individual amino acid differences between Asibi and 17D, grouped by CPER fragment, as well as all hamster adaptation mutations. Variant frequencies are color-coded: yellow indicates >50% Asibi variant, blue indicates >50% 17D variant. Frequencies of hamster adaptation mutations are shown using a red color scale. Grey boxes represent positions with low sequencing coverage (<100 reads). **(C)** Virus production from BHK-21 cells transfected with CPER product, quantified by focus-forming assay (FFU/mL: focus-forming units per mL of supernatant). **(D)** Growth kinetics of recombinant YFVs on Vero cells inoculated with passage-0 stocks (i.e., filtered and titered BHK-21 supernatants) at MOI of 0.1, quantified by focus-forming assay (E-G) 5-7-week-old female hamsters were inoculated intraperitoneally with 1 × 10^5^ FFU of rHA1-Asibi (orange, n = 17), rHA7-Asibi (brown, n = 15), rHA1-17D (light blue, n = 5), or rHA7-17D (dark blue, n = 3). Control groups from Fig 1 are included for comparison. Data represents a composite of 9 independent experiments. **(E)** Survival analysis. **(F)** Body weight at 6 dpi, shown as a percentage of weight at the time of inoculation. **(G)** Viremia measured at 3 dpi, expressed as YFV genome copies per mL of serum (log₁₀). Dashed lines represent the lower limits of detection. Error bars show mean ± SEM. Statistical significance determined via one-way ANOVA with multiple comparisons. In F, comparisons were made to the mock-infected group. In G, rHA1-Asibi and rHA7-Asibi were compared to HA-Asibi, while rHA1-17D and rHA7-17D were compared to 17D. (**: p < 0.01; ****: p < 0.0001; ns: not significant).

To investigate the genetic basis of 17D attenuation, we first developed a circular polymerase extension reaction (CPER)-based reverse genetics system to generate recombinant YFV strains (Fig 2A). This strategy combines genome fragments along with a “linker” plasmid that, during CPER, forms a cDNA-launch vector that can be used to rescue infectious YFV upon transfection into BHK-21 cells (Fig 2A). Using this system we rescued recombinant Asibi or 17D viruses that included the single most important hamster-adapting mutation (E-D155A, referred to as rHA1-Asibi and rHA1-17D) [25] as well as YFVs containing all seven hamster-adapting mutations (referred to as rHA7-Asibi and rHA7-17D). We then used amplicon-based deep sequencing to confirm the identity of all recovered viruses (Fig 2B). Recovery of infectious YFVs from transfected BHK-21 cells, as well as the replication kinetics of passage-zero (p0) virus stocks, were confirmed by focus-forming assay (Fig 2C–2D) and observation of cytopathic effect (S1 Fig).

We then assessed the virulence/attenuation of the rHA-YFVs in hamsters using biological isolates of HA-Asibi and 17D as comparators. For these studies, and all subsequent studies, hamsters were inoculated intraperitoneally with 1 × 10^5^ focus-forming units of sequence-confirmed p0 virus (i.e., clarified supernatant from transfected BHKs). The rHA1-17D and rHA7-17D viruses replicated in hamsters but were fully attenuated, with hamsters exhibiting no significant disease compared to mock-infected hamsters. In contrast, rHA1-Asibi and rHA7-Asibi viruses replicated to higher titers, caused significant weight loss, and resulted in fatality rates of ~6% and ~25%, respectively (Fig 2E–2G). These results demonstrate that recombinant hamster-adapted YFVs reproduce key phenotypes of the biological YFV isolates in hamsters.

### Attenuation determinant(s) of 17D maps to Fragment 2a containing non-structural genes

**Fig 3:**
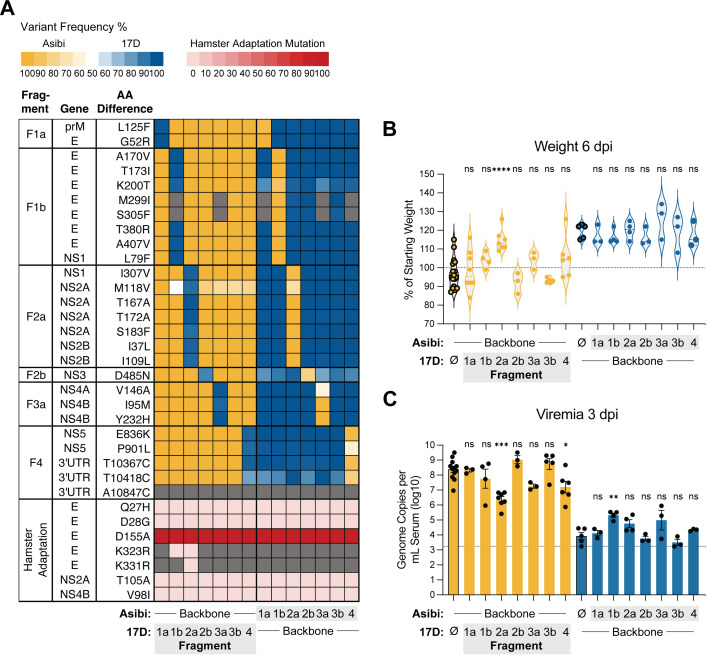
Attenuation determinant(s) of 17D map to Fragment 2a containing non-structural genes. **(A)** Heatmap showing the frequency of Asibi/17D mutations across chimeric rHA1-YFVs in which individual CPER fragments were swapped between Asibi and 17D backbones; see description in Fig 2B for additional details. **(B)** Body weight of hamsters inoculated with 1 × 10⁵ FFU of each virus at 6 dpi, shown as a percentage of of starting weight. Data from animals inoculated with the recombinant parent virus are shown with bolded outlines. **(C)** Viremia measured at 3 dpi, expressed as genome copies per mL of serum (log₁₀). Dashed line in C represents the lower limit of detection. Error bars show mean ± SEM. Statistical significance was determined by one-way ANOVA with multiple comparisons, with Asibi-backbone chimeras compared to rHA1-Asibi, and 17D-backbone chimeras compared to rHA1-17D. (**: p < 0.01; ***: p < 0.001; ****: p < 0.0001; ns: not significant.).

We next sought to determine which specific regions of the YFV genome are responsible for the attenuation phenotype of 17D in the hamster model. Using CPER, we created a panel of recombinant hamster-adapted Asibi/17D chimeric viruses. Each virus contained a single fragment “swapped” from the opposite strain while retaining the remaining six fragments from its original backbone (Figs 2A and 3A). To simplify the number of variables in this approach, we only introduced the E-D155A (e.g., rHA1) hamster-adapting mutation into these chimeric viruses, using 6 dpi weight loss and 3 dpi viremia as readouts of YFV virulence/attenuation. Among viruses with the rHA1-Asibi backbone, only the chimera containing CPER “Fragment 2a” from rHA1-17D exhibited attenuation, whereas all other rHA1-Asibi chimeric viruses remained virulent (Fig 3B–3C). In contrast, none of the rHA1-17D viruses containing Asibi-derived fragments showed increased virulence. These findings suggest that Fragment 2a contains mutation(s) that are necessary for YFV attenuation in the hamster model.

### Attenuation determinant(s) of 17D map to NS2B

**Fig 4:**
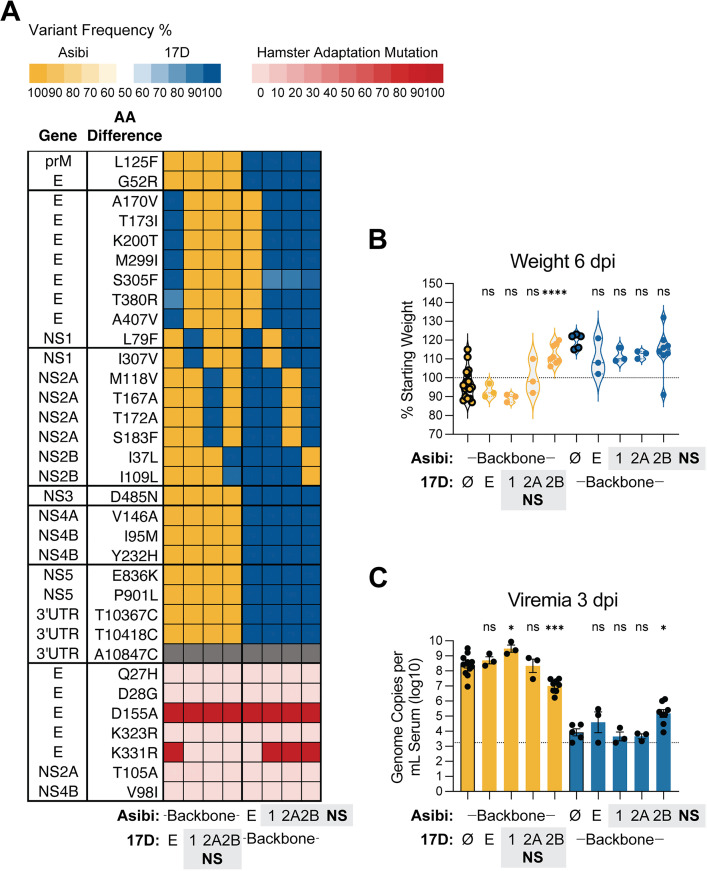
Attenuation determinant(s) of 17D map to NS2B. **(A)** Heatmap showing the frequency of Asibi/17D mutations across chimeric rHA1-YFVs in which individual genes (E, NS1, NS2A, and NS2B) were swapped between Asibi and 17D backbones; see description in Fig 2B for additional details. **(B)** Percent body weight of hamsters inoculated with 1 × 10⁵ FFU of each virus at 6 dpi, shown as a percentage of starting weight. Data from animals inoculated with the recombinant parent virus are shown with bolded outlines. **(C)** Viremia measured at 3 dpi, expressed as genome copies per mL of serum (log₁₀). Dashed line in C represents the lower limit of detection. Error bars show Data represented as mean ± SEM. Statistical significance was determined by one-way ANOVA with multiple comparisons, with Asibi-backbone chimeras compared to rHA1-Asibi, and 17D-backbone chimeras compared to rHA1-17D. (*: p < 0.05; **: p < 0.01; ****: p < 0.0001; ns: not significant.).

Fragment 2a encodes multiple viral proteins harboring mutations that differ between Asibi and 17D, including NS1, NS2A, and NS2B (Fig 2A). Thus, in order to further delineate the contribution of specific viral genes to the observed attenuation phenotype, we generated rHA1-Asibi and rHA1-17D viruses containing NS1, NS2A, and NS2B gene swaps. We also included swaps for the E gene due to the high concentration of 17D-associated mutations in this gene and the historical focus on E as an important determinant of 17D immunogenicity and attenuation [6,15,18,30] (Fig 4A). Among these chimeric viruses, only rHA1-Asibi containing 17D-derived NS2B was attenuated (Fig 4B–4C). Consistent with the Fragment swaps presented in Fig 2, none of the rHA1-17D viruses carrying Asibi-derived genes exhibited increased virulence. However, rHA1-17D with Asibi-NS2B resulted in significantly higher viremia, though this was not associated with significant weight loss by 6 dpi, with the exception of one hamster.

### The NS2B-I109L mutation attenuates rHA1-Asibi

**Fig 5:**
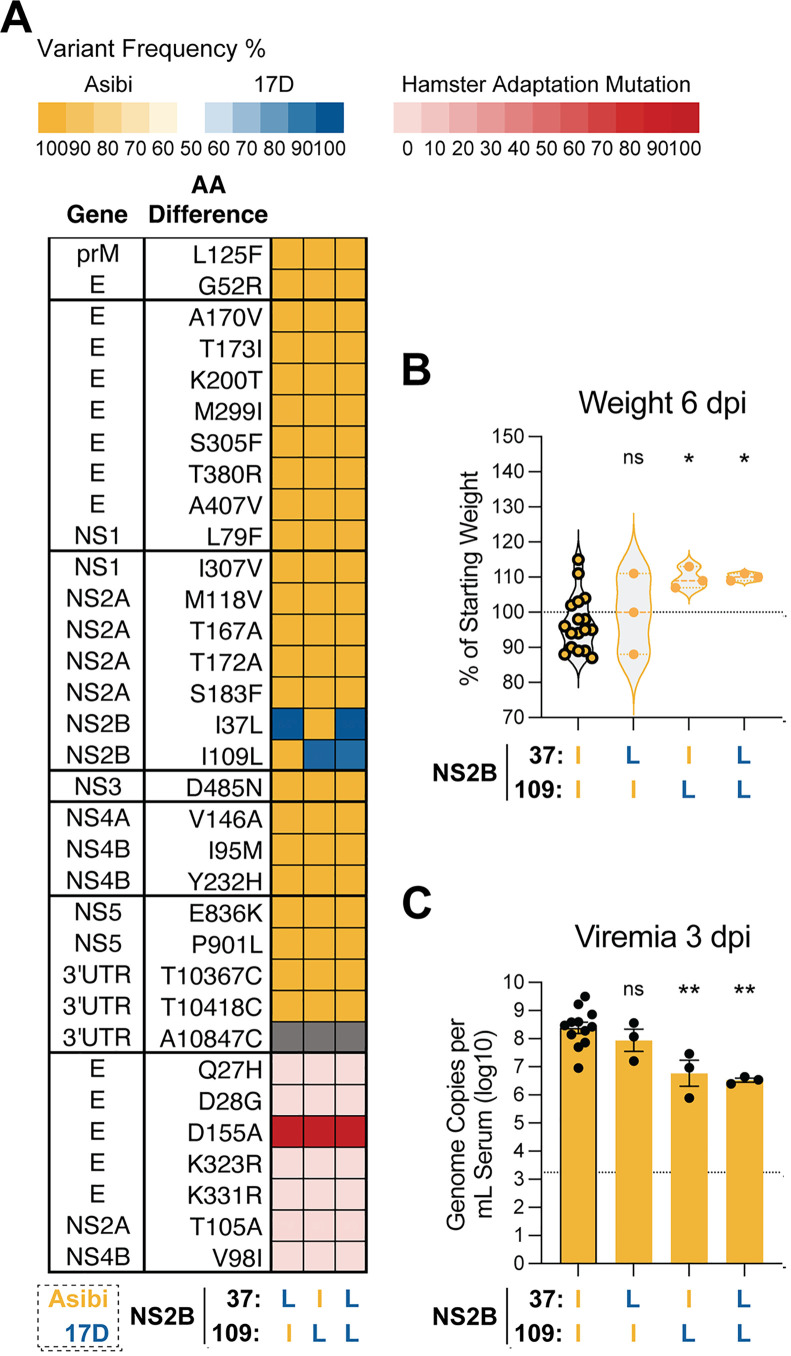
The NS2B-I109L mutation attenuates rHA1-Asibi. (A) Heatmap showing the frequency of Asibi/17D mutations across rHA1-Asibi chimeras in which single or double 17D-NS2B mutations were introduced; see description in Fig 2B for additional details. (B) Percent body weight of hamsters inoculated with 1 × 10^5^ FFU of each virus at 6 dpi, shown as a percentage of starting weight. Data from animals inoculated with the recombinant parent virus are shown with bolded outlines. (C) Viremia measured at 3 dpi, expressed as genome copies per mL of serum (log_10_). Dashed line in C represents the lower limit of detection. Error bars show Data represented as mean ± SEM. Statistical significance was determined by one-way ANOVA with multiple comparisons, with Asibi-backbone chimeras compared to rHA1-Asibi. (*: p < 0.05; **: p < 0.01; ns: not significant.).

Two mutations, both encoding isoleucine-to-leucine substitutions, differentiate 17D from Asibi in NS2B: one at amino-acid position 37 (I37L) and the other at position 109 (I109L). To determine the individual and combined contributions of these mutations to attenuation, we first introduced each mutation alone or together into the rHA1-Asibi backbone (Fig 5A). In this context, I37L alone was non-attenuating, whereas I109L––either alone or in combination with I37L––significantly attenuated the rHA1-Asibi (Fig 5B–5C). This suggests that I109L plays a dominant role in modulating NS2B function in the context of the rHA1-Asibi backbone.

### The NS2B mutations I37L and I109L cooperate to attenuate rHA7-Asibi

**Fig 6:**
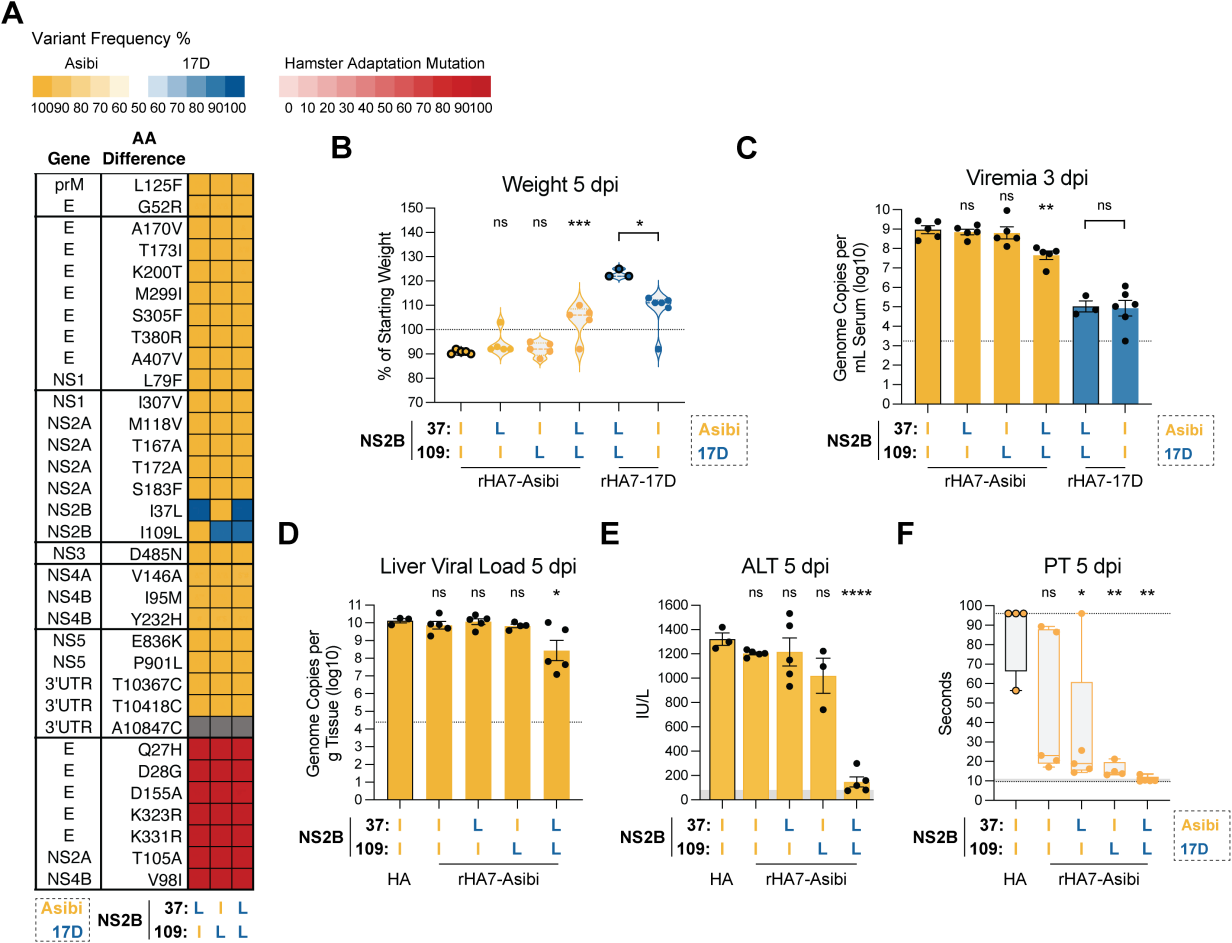
The NS2B mutations I37L and I109L cooperate to attenuate rHA7-Asibi. **(A)** Heatmap showing the frequency of Asibi/17D mutations across rHA7-Asibi chimeras in which single or double 17D-derived NS2B mutations were introduced into rHA7-Asibi; see description in Fig 2B for additional details. **(B)** Percent body weight of hamsters inoculated with 1 × 10⁵ FFU of each virus at 5 dpi, shown as a percentage of starting weight. Data from animals inoculated with the recombinant parent virus are shown with bolded outlines. **(C)** Viremia measured at 3 dpi, expressed as genome copies per mL of serum (log₁₀). Dashed line in C represents the lower limit of detection. Error bars show Data represented as mean ± SEM. **(D)** Liver viral load, (E) alanine aminotransferase (ALT), and (F) prothrombin time (PT) measured at 5 dpi. Dashed lines represent the lower and upper limits of measurement range; the gray shaded area represents the reference range for hamsters, calculated from pooled data of mock-infected animals (see Methods for details). Error bars show Data represented as mean ± SEM. Statistical significance was assessed by one-way ANOVA with multiple comparisons: Asibi-backbone chimeras were compared to rHA7-Asibi in panels B-C, and to HA-Asibi in panels D-F; in B and C, the two rHA7-17D viruses were compared using unpaired t-test (*: p < 0.05; **: p < 0.01; ***: p < 0.001; ****: p < 0.0001; ns: not significant).

We next evaluated the impact of 17D-associated mutations in NS2B in the context of the more virulent rHA7-Asibi backbone (Fig 6A). In this context, neither mutation by itself was sufficient to prevent weight loss or reduce viremia (Fig 6B–6C). However, when supplied in combination, these mutations reduced the serum viral load and prevented weight loss. To study the combined effect of these mutations on rHA7-Asibi pathogenesis in greater detail, we measured liver viral loads (Fig 6D), liver damage, as determined by increases in the concentration of serum alanine transaminase (ALT) (Fig 6E), and coagulopathy, as determined by measuring prothrombin time (PT) (Fig 6F). Viral replication in the liver and liver damage were unaffected by either mutation by itself, but were significantly reduced when the 17D versions (i.e., leucine at 37 and 109) were supplied in combination. Significant reductions in the PT were observed when either mutation was present, again with I109L having a more pronounced effect. Nevertheless, full restoration of the PT to normal was only observed when the mutations were present together.

## Discussion

Since the creation of the attenuated YFV strain 17D in the 1930s and its widespread adoption as a vaccine for YF, understanding the mechanism by which 17D elicits a robust immune response without causing disease has been an important yet elusive goal in virology research [31]. Conceptually, this can be further divided into two broad features that render 17D highly effective as a vaccine: enhanced immunogenicity and reduced virulence (a.k.a., attenuation)––though the two are likely interrelated. Studies of 17D in human vaccine recipients have largely focused on characterizing the immunogenicity of 17D, demonstrating that 17D elicits a robust innate immune response that aids in the development of a polyfunctional and durable T cell response [32–35]. However, an inherent limitation of these studies is the lack of comparison to infection with virulent wild-type YFV (e.g., Asibi), which can only be performed in an animal model but is ultimately important for understanding the mechanistic basis of 17D attenuation. Towards this goal, one study compared transcriptional responses in peripheral blood of macaque monkeys infected with 17D versus the YFV-DakH1279 strain––a West African lineage similar to Asibi––revealing that 17D induces a transcriptional profile largely restricted to induction of the immune genes while YFV-DakH1279 infection resulted in transcription of a larger set of genes with signatures of a uncoordinated/dysregulated immune response [36]. Studies of 17D in mice have shown that interferon (IFN) type I, II, and III contribute to control of 17D replication [37–39], but the relevance of these studies to 17D attenuation in humans remains unclear given the natural resistance of the murine host to the development of hepatotropic/viscerotropic disease. Thus, in vivo studies of 17D attenuation have focused almost exclusively on the host, without clearly defining virus-intrinsic features of attenuation.

In vitro studies have allowed for more mechanistic comparisons between 17D and Asibi, most of which have focused on the Envelope (E) glycoprotein that coats the surface of the virion [6]. Structurally, the conformation of E on 17D appears to present unique antibody epitopes that generate more robust neutralizing antibody response compared to E of Asibi or other wild-type YFV strains [30,40]. The 17D mutations in E also appear to have functional consequences, resulting in enhanced binding to glycosaminoglycans and the adoption of a clathrin-independent entry pathway––both of which which may alter cellular tropism and dissemination, though the importance of these features to 17D immunogenicity and attenuation in vivo remain to be further characterized. Infection of various cell lines and primary cell types by 17D and Asibi have provided somewhat conflicting results, but the emerging consensus is that 17D induces a significantly greater antiviral response than Asibi, and this response appears to be primarily driven by increased production of type I interferon (IFN). Nevertheless, the cellular process(es) by which 17D facilitates a more robust IFN response remains unclear. Given the prior focus on E as a key determinant of 17D immunogenicity and attenuation, our mapping of attenuating mutations to NS2B is unexpected and creates new opportunities for further understanding the multiple mutations (and mechanisms) that render 17D effective as a vaccine.

NS2B serves as a cofactor for the NS3 protease, forming the NS2B-NS3 (NS2B3) protease complex that is crucial for viral polyprotein processing and viral replication. The complex cleaves the viral polyprotein at multiple junctions, and one possible mechanism by which NS2B mutations attenuate 17D could be related to reduced cleavage efficiency, which would ultimately slow the speed of viral replication allowing more time for the mounting of a favorable immune response. Another non-mutually-exclusive mechanism may involve alterations to the ability of NS2B to antagonize host innate immunity. Recent work has demonstrated that NS2B3 of multiple flaviviruses (ZIKV, DENV, and WNV) cleave the human stimulator of interferon genes (STING) protein, a critical activator of the interferon (IFN) signaling pathway [41]. While the NS2B3 of YFV has not been shown to cleave human STING, cleavage of STING or other host molecules involved in the IFN response would be consistent with studies showing that 17D induces stronger type I IFN responses compared to Asibi. Thus, it is conceivable that 17D mutations in NS2B attenuate the virus by slowing the efficiency of viral polyprotein cleavage and/or reducing the ability of NS2B to counteract the host immune response. If true, such a mechanism would put individuals with undiagnosed innate immune deficiencies at high risk for adverse reactions to the 17D vaccine, potentially explaining the rare but well-described YF-like disease caused by 17D in a small fraction of vaccinees [42,43].

Regardless of the mechanism by which mutations in NS2B ultimately alter its function to result in YFV attenuation, it seems noteworthy that these two isoleucine-to-leucine substitutions are highly conservative amino acid changes––both contain small aliphatic R groups embedded in separate transmembrane domains––which would not be expected to dramatically alter the structure of NS2B. However, even small perturbations in transmembrane domains can impact ectodomain orientation and function particularly if there are interprotein interactions, such as in the case of NS2B interacting with NS3. The fact that both mutations are required for full attenuation of the virulent rHA7-Asibi suggests that these residues cooperate to alter NS2B function. As such, the acquisition of both NS2B mutations during the passaging process in the 1930s was likely a low-probability event, potentially explaining why Asibi did not become attenuated when the Theiler laboratory repeated the entire passaging experiment a second time [44]. Given the central role that 17D has since played in combating YF and as a model immunogen, it is remarkable to consider its origins as a low probability “happy accident” due to the chance near-simultaneous accumulation of these two mutations. This also raises the possibility that 17D is less attenuated than currently assumed. While minimal attenuation could conceivably be important for generating a robust immune response, this also has important implications for vaccine safety. Thus, as we continue to learn more about the determinants of 17D immunogenicity and attenuation, it remains to be seen whether the safety of the 17D platform can be enhanced through the engineering of additional features of attenuation without compromising immunogenicity.

In summary, our identification of two mutations in NS2B that cooperate to attenuate 17D is a step towards understanding the genetic basis of this important live-attenuated vaccine. One caveat to this finding may be the possibility that it is specific to the hamster host and thus has limited applicability to attenuation of 17D in humans and nonhuman primates. While followup studies in these “natural” hosts may be needed to confirm these findings, it is worth noting that our approach would have been technically and financially challenging in the gold-standard nonhuman primate model. Thus, this discovery opens new avenues to further understand the mechanistic basis of YFV virulence and attenuation, with implications for the pathogenesis of (and development of vaccines against) other *Orthoflaviviruses*.

## Materials and methods

### Ethics statement

All animal experiments were conducted in compliance with NIH and UW-Madison policies and procedures, and were approved by the Institutional Animal Care and Use Committee (IACUC protocol # M006443) and the Institutional Biosafety Committee (IBC protocol # B00000929).

### Cell lines

BHK-21 and Vero-WHO cells were maintained in Dulbecoo’s Modified Eagle Medium (DMEM; Gibco) supplemented 5% fetal calf serum (FCS, Omega) at 37^o^C with 5% CO_2_.

### Viruses

Wild-type YFV-Asibi was obtained from the World Reference Center for Emerging Viruses and Arboviruses (University of Texas Medical Branch, Galveston, Texas). YFV-17D-204 and Asibi p7 strain were kindly provided by Dr. Jorge Osorio (UW-Madison) and Dr. Alan Barrett (University of Texas Medical Branch, Galveston, Texas), respectively. A stock of hamster-adapted Asibi (HA-Asibi) was prepared by diluting serum collected at 4 dpi after inoculation of a hamster with Asibi p7. Virus stocks were quantified by focus-forming assay and stored at -80^o^C. Experiments involving wild-type and hamster-adapted yellow fever viruses were performed in ABSL-3 at University of Wisconsin-Madison by vaccinated personnel.

### Hamsters

Syrian golden hamsters (Charles River), female and 5–7 weeks of age, were randomized by veterinary staff upon arrival and housed in a controlled environment with regulated light/dark cycles, temperature, and ad libitum access to food and water in ABSL-3. Viral inoculations were performed via intraperitoneal (i.p.) injection of 100 µL containing 1 × 10^5^ focus-forming units (FFU) of virus diluted in tissue culture media (DMEM+2%FCS). Survival, weight, and clinical signs of disease were monitored daily. Receiver operating characteristic (ROC) curve analysis of pilot survival studies showed that any animal with a clinical score of ≥8 would not survive, and this cutoff was used as criteria for euthanasia in subsequent studies. Blood was collected via gingival vein or via direct cardiac cannulation at the time of euthanasia. For euthanasia, animals were deeply sedated with ketamine via i.p. injection followed by thoracotomy.

### RNA extraction

RNA was extracted from tissue homogenates, serum, and supernatant using the MagMAX Viral Isolation Kit (Thermo Fisher, AMB1836–5) on the KingFisher Flex System (Thermo Fisher). For tissue homogenates, animals were sedated and the cardiovascular tree was perfused with 50 mL of sterile saline at the time of euthanasia. Tissues were then collected and snap-frozen. Tissue homogenization was performed by bead-beating in DMEM+2%FCS using the Beadblaster-24 (Benchmark). RNA was then extracted from tissue/media homogenate and quantitative results were normalized to the pre-homogenization tissue weight. For serum and cell culture supernatant, carrier RNA was used during RNA extraction and quantitative results were normalized to the starting volume (typically 20μL).

### YFV reverse genetics

The YFV genome was randomly divided into seven fragments of approximately 1500 bp. Sequence-specific primers (S1 Table) were designed accordingly and cDNA was synthesized and amplified using the SuperScript IV One-Step RT-PCR System (Invitrogen, 12594025). To prevent potential batch variations introduced by RT-PCR-based fragment amplification, each of the seven cDNA fragments was cloned into a shuttle vector via Gibson Assembly (NEB, E5520). A linker plasmid containing the bovine growth hormone (BGH) polyadenylation signal, hepatitis delta virus (HDV) ribozyme, and cytomegalovirus (CMV) enhancer and promoter was generated based on a construct kindly provided by Dr. Susan Baker (Loyola University Chicago), originally developed by Dr. Michaela Gack (Lerner Research Institute, Cleveland Clinic Florida). The assembled plasmids were transformed into NEB5-alpha competent cells (NEB, C2987) and successful assembly was verified by colony PCR. Plasmid DNA was purified using the QIAprep Spin Miniprep Kit (Qiagen, 27104). Plasmid integrity was confirmed by Oxford Nanopore Technologies-based whole plasmid sequencing (Plasmidsaurus). If point mutations were needed (e.g., hamster-adapting mutations), site-directed mutagenesis was performed using the Q5 Site-Directed Mutagenesis Kit (NEB, E0552).

Amplicons for CPER were then generated using each sequence-confirmed plasmid as template (1ng per 50μL reaction) and the PrimeSTAR GXL DNA polymerase (Takara Bio, R050A) with the following cycling program: 30 cycles of 98^o^C for 10 seconds, 60^o^C for 15 seconds, and 68^o^C for 2 minutes. Primer sequences for this reaction can be found in S2 Table. Amplified fragments were gel purified (Qiagen, 28506), phosphorylated (NEB, M0201), cleaned up (Qiagen, 28506) again, and assembled into a circularized full-length YFV cDNA product via CPER with the linker sequence connecting the YFV 5′ and 3′ UTRs. The CPER reaction was performed using PrimeSTAR GXL DNA polymerase with an optimized cycling program: initial denaturation at 98^o^C for 2 minutes, followed by 35 cycles of 98^o^C for 10 seconds, 55^o^C for 15 seconds, and 68^o^C for 12 minutes, with a final extension at 68^o^C for 15 minutes. For a 50μL reaction, 0.05 pmoles of each fragment was used and transfected into a well of a 6-well plate; for larger reactions, reagent input was scaled accordingly (e.g., 400μL reaction, split equally between four PCR tubes, for transfection into a T75 flask). The CPER product was stored at 20^o^C until transfection into BHK-21 cells for virus recovery.

### Recombinant virus recovery and stock preparation

The CPER product was transfected into BHK-21 cells using TransIT-X2 transfection reagent, following the manufacturer’s protocol (Mirus Bio, MIR 6004). Cells were monitored for cytopathic effects (S1 Fig), and supernatants were collected at 4–5 days post transfection when cytopathic effect was observed in 70–100% of the culture. Supernatants were clarified by centrifugation at 1000g for 5 minutes, then passed through a 0.45μm filter and aliquoted to create a “stock” for each recombinant virus. Viral stock infectious titers were quantified using focus-forming assay (description below).

### Viral growth kinetics

To assess viral replication kinetics, target cells were inoculated with YFV or recombinant YFV at a multiplicity of infection (MOI) of 0.1 in inoculation medium (DMEM with 2% FCS). After one hour of adsorption, the inoculum was removed, cells were washed with PBS, and fresh medium was added. Supernatants were collected at 24-hour intervals for five days and stored at -80^o^C. Viral titers at each time point were determined by focus-forming assay.

### Focus-forming assay

Vero-WHO cells (ATCC) were seeded at 5 × 10⁴ cells per well in a 96-well plate and incubated overnight. Serial dilutions of virus-containing samples were added and incubated for 1 hour at 37°C with rocking. A 2% methylcellulose overlay was applied, and plates were incubated for 48 hours before fixation with 4% paraformaldehyde. Cells were permeabilized with saponin-based buffer and stained with anti-YFV 2D12 monoclonal antibody (1:800) overnight at 4°C. The antibody was prepared from clarified and filtered supernatant collected from mouse hybridoma cells (ATCC, CRL-1689). After washing, cells were incubated with an HRP-conjugated secondary antibody (1:1000, Jackson ImmunoResearch) and developed with TrueBlue peroxidase substrate (Seracare, 5510–0030). Foci were scanned and quantified using a Biospot plate reader (Cellular Technology).

### Viral genome sequencing

Amplicons tiling the YFV genome were designed in PrimalScheme using a 250 bp amplicon length and two pools, taking care to avoid primer binding at variant sites within 17D, Asibi, and HA-Asibi. Illumina adaptors were appended to the 5′ end of each primer (F:ACACTCTTTCCCTACACGACGCTCTTCCGATCT; R: GTGACTGGAGTTCAGACGTGTGCTCTTCCGATCT). Primers were synthesized in pooled format (oPools, IDT). Note that after initial testing of this PrimalScheme, additional primer-sets were spiked into the oPools to boost coverage in low-coverage areas (seen in Fig 2). The final set of PrimalScheme primers can be found in S3 Table. RNA was extracted from supernatant as described above, and amplicons were generated using the SuperScript IV One-Step RT-PCR System (Invitrogen, 12594025). Each sample was amplified in two separate reactions using two primer pools, and thermocycling was carried out with reverse transcription at 48^o^C for 30 minutes and initial denaturation at 98^o^C for 2 minutes, followed by 37 cycles of 98^o^C for 15 seconds, 60^o^C for 3 minutes, and 72^o^C for 20 seconds, with a final extension at 72^o^C for 5 minutes. Amplified products from both reactions were pooled and purified using the QIAquick PCR & Gel Cleanup Kit (Qiagen, 28506). PCR products were submitted to the UW-Madison Biotechnology Center for index PCR and library preparation using the Illumina TruSeq system. Sequencing was performed on an Illumina NovaSeq platform. Paired-end fastq reads were processed in Geneious Prime v2022.2.2, with normalization and error correction using the BBnorm plugin set to target coverage depth of 1000. Reads were then trimmed by 30 nt at the 5′ and 3′ ends and mapped to the YFV reference genomes (YFV-Asibi: AY640589; YFV-17D: MN708488) with Bowtie2. Variant analysis was conducted using “Find Variations/SNPs” function in Geneious, using a minimum variant frequency of 5%. Variant tables were then exported to excel and a color-coded heatmap was overlaid.

### Quantitative YFV viral load by RT-PCR

RNA extraction was performed as described above. RT-qPCR was performed using the TaqMan RNA-to-CT 1-Step Kit (Thermo Fisher, 4392653) on the QuantStudio 6 Pro Real-Time PCR System (Thermo Fisher) under the following cycling condition: 1) 48^o^C for 15 min, 2) 95^o^C for 10 min, 3) 95^o^C for 15 sec, 4) 60^o^C for 1 min, and 5) repeat from step 3 for additional 49 cycles. A primer-probe set for RT-qPCR (IDT) was designed that targets the 5’ UTR of YFV: forward primer (5’-AGGTGCATTGGTCTGCAAAT-3’), reverse primer (5’-TCTCTGCTAATCGCTCAAIG-3’), and probe (5’-/56-FAM/GTTGCTAGGCAATAAACACATTTGGA/3BHQ_1/-3’) and published previously [45]. An RNA standard was developed from a pCR-Blunt-based construct (Invitrogen) that contains an 84-bp fragment from the 5’UTR region of YFV with the binding sites of the primers and probe (5’-AGGTGCATTGGTCTGCAAATCGAGTTGCTAGGCAATAAACACATTTGGATTAATTTTAATCGTTCGTTGAGCGATTAGCAGAGA-3’). The construct was linearized with restriction enzyme digestion and *in vitro* transcription (MEGAscript T7 Transcription Kit, Invitrogen, AM1334) was performed. The transcript was purified (MEGAclear Transcript Clean-Up Kit, Invitrogen, AM1908), quantified via spectrophotometry (NanoDrop), and diluted to 10^10^ copies/µL. Ten-fold dilutions of the transcript, ranging from 10^8^ to 10 copies/reaction, were used as a standard curve.

### Serum ALT assay

Serum ALT level was quantified using a commercial ALT reagent kit (Teco Diagnostics, A526-120) according to the manufacturer’s instructions with modification to a 96-well format. Briefly, 10 µL of serum was mixed with 50 µL of ALT substrate and incubated at 37°C for 30 minutes. 50 µL was added to the mixture. After a 10-minute incubation at 37°C, 200 µL of ALT color developer was added, followed by a 5-minute incubation at 37°C. The plate was read on a plate reader (CLARIOstar, BMG Labtech) at 505 nm. The hamster reference range (7.3 - 76.29 IU/L) was established using pooled data from 8 mock-infected hamsters, based on the calculated upper and lower bounds.

### Prothrombin time

Prothrombin time was measured from whole blood using the CoaguChek XS System (Roche) according to the manufacturers’ instructions. The hamster “reference” range (9.6 - 11 seconds) was established using pooled data from 13 mock-infected hamsters, based on the calculated upper and lower bounds.

## Supporting information

S1 Fig Cytopathic effects in BHK-21 cells following transfection with YFV CPER products. Whole Asibi or 17D CPER products from 50 µL reactions were transfected into BHK-21 cells seeded in a 6-well plate format. For comparison, cells were inoculated with Asibi or 17D-204 at a multiplicity of infection (MOI) of 0.01. Cytopathic effects (CPE) were monitored daily for 7 days post inoculation or transfection (dpi/t).
(DOCX)

S1 Table Primers used for RT-PCR of fragments from YFV genome. (DOCX)

S2 Table Primers used for generation of fragments for YFV CPER. (DOCX)

S3 Table YFV deep sequencing primers designed using primal scheme. (DOCX)
