# Supplementary material for: Two mutations in NS2B are responsible for attenuation of the yellow fever virus (YFV) vaccine strain 17D
Source: PLoS Pathog. 2025 Jul 31;21(7):e1013373. doi: 10.1371/journal.ppat.1013373 (PMC12312905; doi:10.1371/journal.ppat.1013373)
Supplement: S1 Fig — Whole Asibi or 17D CPER products from 50 µL reactions were transfected into BHK-21 cells seeded in a 6-well plate format. For comparison, cells were inoculated with Asibi or 17D-204 at a multiplicity of infection (MOI) of 0.01. Cytopathic effects (CPE) were monitored daily for 7 days post inoculation or transfection (dpi/t). (DOCX) [file ppat.1013373.s001.docx]

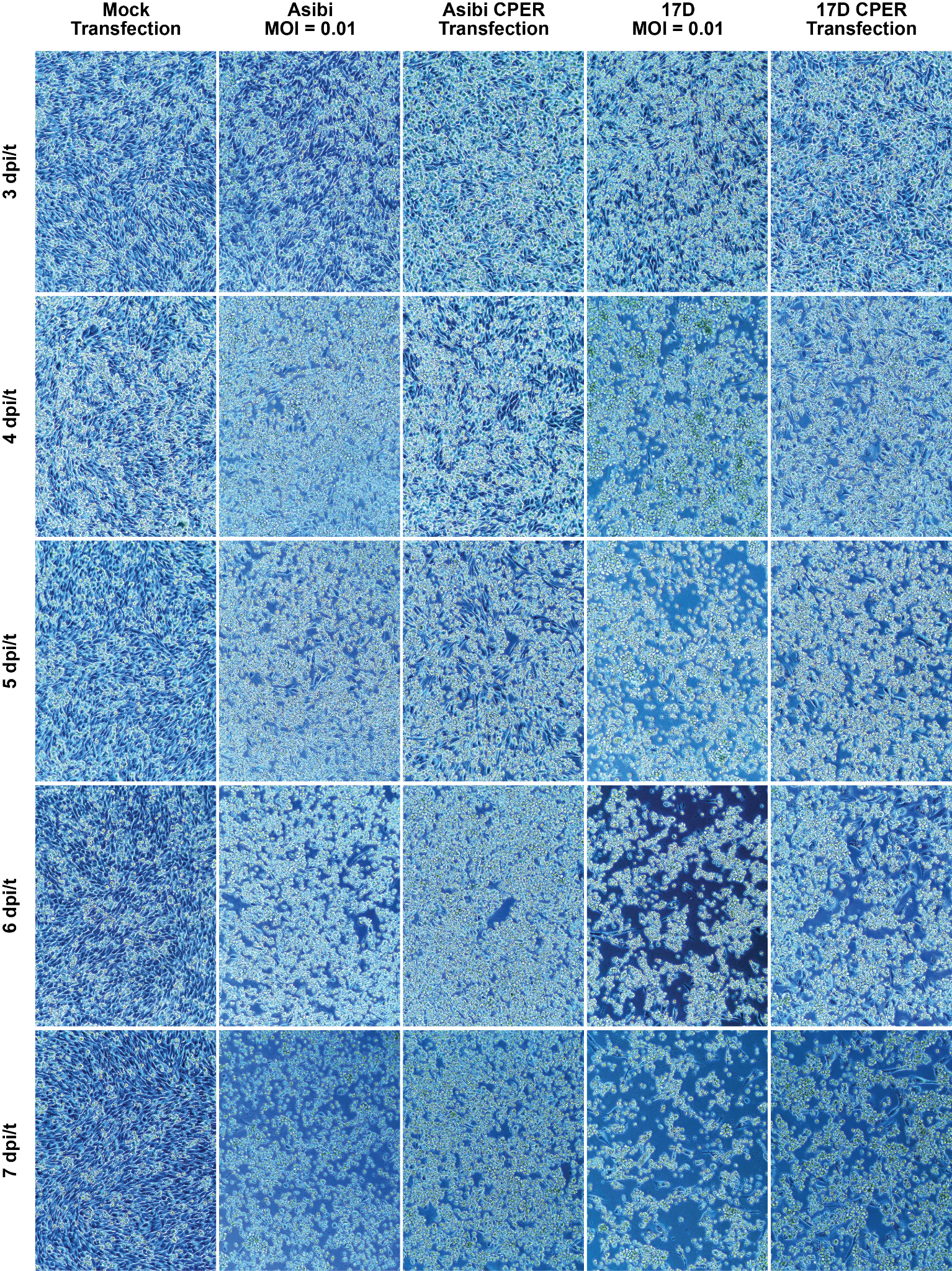


**S1 Fig. Cytopathic effects in BHK-21 cells following transfection with YFV CPER products.** Whole Asibi or 17D CPER products from 50 µL reactions were transfected into BHK-21 cells seeded in a 6-well plate format. For comparison, cells were inoculated with Asibi or 17D-204 at a multiplicity of infection (MOI) of 0.01. Cytopathic effects (CPE) were monitored daily for 7 days post inoculation or transfection (dpi/t).
