# Supplementary material for: Two mutations in NS2B are responsible for attenuation of the yellow fever virus (YFV) vaccine strain 17D
Source: PLoS Pathog. 2025 Jul 31;21(7):e1013373. doi: 10.1371/journal.ppat.1013373 (PMC12312905; doi:10.1371/journal.ppat.1013373)
Supplement: S1 Table — (DOCX) [file ppat.1013373.s002.docx]

| **S1 Table. Primers used for RT-PCR of fragments from YFV genome** | |
| --- | --- |
| **Name** | **Sequence** |
| pET28a-YFVFrag1-F1 | CATTATCGCCGGCATGGCGGCCCCACGGGTGCGCATAGTAAATCCTGTGTGCTAATTGAGGTG |
| pET28a-YFVFrag1a-R1 | GGGTCCTCAACGACAGGAGCACGATCATGCGCACTCGGTATTCCAATTTTCCTGCTTGG |
| pET28a-YFVFrag1b-F1 | CATTATCGCCGGCATGGCGGCCCCACGGGTGCGCATCATTAAGACTCTCAAGTTTGATGCCCTG |
| pET28a-YFVFrag1-R1 | GGGTCCTCAACGACAGGAGCACGATCATGCGCACGATCCATCGCAGTCTATGGTGTATTCAAAG |
| pET28a-YFVFrag2-F1 | CATTATCGCCGGCATGGCGGCCCCACGGGTGCGCATTATCTTGGGTGCAGCGGTGAAC |
| pET28a-YFVFrag2a-R1 | GGGTCCTCAACGACAGGAGCACGATCATGCGCACCTTGTGACATGCCACATTGTGTG |
| pET28a-YFVFrag2b-F1 | CATTATCGCCGGCATGGCGGCCCCACGGGTGCGCATAGGAGCTTTCCTTGTCAGGAATGG |
| pET28a-YFVFrag2-R1 | GGGTCCTCAACGACAGGAGCACGATCATGCGCACGCCCTGCACTTCACTGTTTCAC |
| pET28a-YFVFrag3-F1 | CATTATCGCCGGCATGGCGGCCCCACGGGTGCGCATTCCTGGAGGAGCAAAGAAGCC |
| pET28a-YFVFrag3a-R1 | GGGTCCTCAACGACAGGAGCACGATCATGCGCACGCCGTATCACGATCCACCTC |
| pET28a-YFVFrag3b-F1 | CATTATCGCCGGCATGGCGGCCCCACGGGTGCGCATACGCAGGCATTTGGCCG |
| pET28a-YFVFrag3-R1 | GGGTCCTCAACGACAGGAGCACGATCATGCGCACTTCCCTGGAAGCCCAATGGTC |
| pET28a-YFVFrag4-F1 | CATTATCGCCGGCATGGCGGCCCCACGGGTGCGCATAACTCAGGAGGAGGAGTGGAAGGC |
| 17D-HDV-R1 | gtcccattcgccattaccgaggggacggtcccctcggaatgttgcccagccggcgccagcgaggaggctgggaccatgccggccAGTGGTTTTGTGTTTGTCATCCAAAGGT |
| Asibi-HDV-R1 | gtcccattcgccattaccgaggggacggtcccctcggaatgttgcccagccggcgccagcgaggaggctgggaccatgccggccAGTGGTTTTGTGTTTTTCATCCAAAGGT |
| pET28a-HDVr-R1 | GGGTCCTCAACGACAGGAGCACGATCATGCGCACgtcccattcgccattaccgag |
| pET28a-linker-F1 | CATTATCGCCGGCATGGCGGCCCCACGGGTGCGCATGGCCGGCATGGTCCC |
| pET28a-CMVlinker-R1 | GGGTCCTCAACGACAGGAGCACGATCATGCGCACACGGTTCACTAAACGAGCTCTG |

Note: For cloning frag4 into pET28a, differences between Asibi and 17D at the 3' end complicated the design. Therefore, we first used strain-specific primers (Asibi-HDV-R1 and 17D-HDV-R1) to add HDVr to the 3'end of Frag4, and then cloned Frag4-HDVr into pET-28a(+) using pET28a-YFVFrag4-F1 and pET28a-HDVr-R1.
