# Supplementary material for: Two mutations in NS2B are responsible for attenuation of the yellow fever virus (YFV) vaccine strain 17D
Source: PLoS Pathog. 2025 Jul 31;21(7):e1013373. doi: 10.1371/journal.ppat.1013373 (PMC12312905; doi:10.1371/journal.ppat.1013373)
Supplement: S2 Table — (DOCX) [file ppat.1013373.s003.docx]

| **S2 Table. Primers used for generation of fragments for YFV CPER** | |
| --- | --- |
| **Name** | **Sequence** |
| linker for CPER-F1 | gcgaatgggacccgctgatcagc |
| CMVlinker for CPER-R1 | CACACAGGATTTACTACGGTTCACTAAACGAGCTCTG |
| CMV-YFVFrag1a for CPER-F1 | CGTTTAGTGAACCGTAGTAAATCCTGTGTGCTAATTGAGGTGCA |
| YFVFrag1a for CPER-R1 | TTGAGAGTCTTAATGTCGGTATTCCAATTTTCCTGCTTGGC |
| HAYFVFrag1a for CPER-R1 | TTGAGAGTCTTAATGGCGGTATTCCAATTTTCCTGCTTGGC |
| YFVFrag1b for CPER-F1 | AAATTGGAATACCGACATTAAGACTCTCAAGTTTGATGCCCT |
| HAYFVFrag1b for CPER-F1 | AAATTGGAATACCGCCATTAAGACTCTCAAGTTTGATGCCCT |
| YFVFrag1b for CPER-R1 | GCACCCAAGATAGATCCATCGCAGTCTATGGTGTATTCA |
| YFVFrag2 for CPER-F1 | CTGCGATGGATCTATCTTGGGTGCAGCGGTGAAC |
| YFVFrag2a for CPER-R1 | GGAAAGCTCCTCTTGTGACATGCCACATTGTGTG |
| YFVFrag2b for CPER-F1 | CATGTCACAAGAGGAGCTTTCCTTGTCAGGAAT |
| YFVFrag2 for CPER-R1 | CTCCTCCAGGAGCCCTGCACTTCACTGTTTCAC |
| YFVFrag3a for CPER-F1 | AAGTGCAGGGCTCCTGGAGGAGCAAAGAAGCCTCTGC |
| YFVFrag3a for CPER-R1 | AATGCCTGCGTGCCGTATCACGATCCACCTCC |
| YFVFrag3b for CPER-F1 | CGTGATACGGCACGCAGGCATTTGGCCGAAG |
| YFVFrag3b for CPER-R1 | TCCTCCTGAGTTTTCCCTGGAAGCCCAATGG |
| YFVFrag4-HDVr for CPER-F1 | GCTTCCAGGGAAAACTCAGGAGGAGGAGTGGAAGGC |
| YFVFrag4-HDVr for CPER-R1 | ctgatcagcgggtcccattcgccattaccgagg |
