# Supplementary material for: Two mutations in NS2B are responsible for attenuation of the yellow fever virus (YFV) vaccine strain 17D
Source: PLoS Pathog. 2025 Jul 31;21(7):e1013373. doi: 10.1371/journal.ppat.1013373 (PMC12312905; doi:10.1371/journal.ppat.1013373)
Supplement: S3 Table — (DOCX) [file ppat.1013373.s004.docx]

| **S3 Table. YFV Deep Sequencing Primers Designed Using Primal Scheme** | | | | | | | |
| --- | --- | --- | --- | --- | --- | --- | --- |
| **Name** | **Sequence** | **Name** | **Sequence** | **Name** | **Sequence** | **Name** | **Sequence** |
| YFVprimal_1F | ACATGTCTGGTCGTAAAGCTCAG | YFVprimal_1R | CCTTAGAACAGCCAAGCCTTGT | YFVprimal_2F | TGTTCAACATTTTGACTGGAAAA | YFVprimal_2R | TTTTCCCGAGGTCCTCAGATGT |
| YFVprimal_3F | TGTTGATGACGGGTGGAGTGA | YFVprimal_3R | CCTGCTGAGTCACACTTACCAT | YFVprimal_4F | CAGATGACATTGATTGCTGGTGC | YFVprimal_4R | CACAAGGTAGGCAATGGTCAGA |
| YFVprimal_5F | GCAACTCCAAAAGATTGAGAGATGG | YFVprimal_5R | GGGCCATAACAGTGACACACTT | YFVprimal_6F | GGATTTCATTGAGGGGGTGCAT | YFVprimal_6R | TTGCACGCATTGTCCCCTTC |
| YFVprimal_7F | AGATTAATGACAAGTGCCCCAGC | YFVprimal_7R | CAATTTTCCTGCTTGGCCCCTA | YFVprimal_8F | GAGTTTGTTTGAGGTTGATCAGACC | YFVprimal_8R | CCCACTGTCTGTCCACTATCCA |
| YFVprimal_9F | CAAACTGCGGTGGACTTTGGTA | YFVprimal_9R | TCCTTTGTAACCCTCATTGCGC | YFVprimal_10F | ACTATCAGAGTACTGGCCCTGG | YFVprimal_10R | TCACCTGCATCACAACAGTG |
| YFVprimal_11F | CACTCAAGGGGACATCCTACA | YFVprimal_11R | CCCAACGATAATGTAGCTGTCTCC | YFVprimal_12F | CCATCGCCTCAACCAATGATGA | YFVprimal_12R | CGTATGAATTCCTTTCCCAACCG |
| YFVprimal_13F | GGAAAGTTGTTCACTCAGACCA | YFVprimal_13R | GACAAAAACATCATGATCACTCCTACC | YFVprimal_14F | CATCATGGGGGCGGTACT | YFVprimal_14R | GAGGCTTTCACTATTGATGCAAGC |
| YFVprimal_15F | AGAGACTCTGATGACTGGCTGA | YFVprimal_15R | CCCGAATTCTGGAAAATGGATGAG | YFVprimal_16F | CAGGGCAGATGAGATCAATG | YFVprimal_16R | CTCCCGTCCCAAACTCCTCTAT |
| YFVprimal_17F | GTCCAGGAAAGAATGCCCGTT | YFVprimal_17R | TGTAATCTAATGCCTCCAAGGTGTG | YFVprimal_18F | TGCAGCGGTGAACGGAAAAA | YFVprimal_18R | ATCCAAGGTCCGTTCGTCTGA |
| YFVprimal_19F | AGATCAATCGGAGGCCCAGTTA | YFVprimal_19R | ACCCATCACTACCATGGAAGCT | YFVprimal_20F | ACCAGATCCACCACGGATAG | YFVprimal_20R | CTCCTCCAACCAACATTTGC |
| YFVprimal_21F | CATGCTGTCCCTTTTGGTTTGG | YFVprimal_21R | GCAGCCCTGGTCTGATTGAAA | YFVprimal_22F | GCTGTGGGATTGCATTTCCATG | YFVprimal_22R | GCATTTATTGTCAGGATGCAG |
| YFVprimal_23F | GCCATGGTGGAGATTGCCTT | YFVprimal_23R | TCTTCTGCATGGAGGTGTCCTT | YFVprimal_24F | ATGGCTGAGGTGAGACTTGC | YFVprimal_24R | CTGAAAAGCCAGTCCTGCCA |
| YFVprimal_25F | GAGTATCCCAGTGAATGAGGCA | YFVprimal_25R | CCTTGTTCACTGAGTGCCACAT | YFVprimal_26F | GAAGCTTGGTGAAGTTTCATGGG | YFVprimal_26R | ACAAGACATCCCCACTTCTCCT |
| YFVprimal_27F | TCTTCTGCTGGTCCTTGCTG | YFVprimal_27R | CAACTTCTTGCCATTCCTGACAAG | YFVprimal_28F | ATTCCAGTCAACCTTCTTGGGG | YFVprimal_28R | GTTGACCACGTTCTTTCCTGGA |
| YFVprimal_29F | AAGTTGGAAGGCAGATGGGATG | YFVprimal_29R | ATATGGCGGACACGAAGGAGT | YFVprimal_30F | TCTTGACTATCCGAGTGGCACT | YFVprimal_30R | ACGTCTTGTCTTCCCAGCTC |
| YFVprimal_31F | TCCCGACAATGCTAAAGAAA | YFVprimal_31R | CTGTAAGTTAGGGTGGCATGGC | YFVprimal_32F | ACACAGGCTTTTTCCGCTCA | YFVprimal_32R | GGAAATTCATCACTAGTCCCAGGC |
| YFVprimal_33F | GCACAGAGCTAGGGCAAATGAA | YFVprimal_33R | ACCACACTCTTTCCAGCCTTAC | YFVprimal_34F | GCATGGTTCCTTCCATCCATCA | YFVprimal_34R | TATTGCCACCTTCCTCCCTTCA |
| YFVprimal_35F | AATGGGAGCCAACCTTTGCG | YFVprimal_35R | TTGTCCAAGAGCATTGAGGCC | YFVprimal_36F | GGAGACTCATACTACTATTCTGAGCC | YFVprimal_36R | CACTTGCCACGAAAGCCAAA |
| YFVprimal_37F | TGAGGGATGACCAGAGGAAAGT | YFVprimal_37R | AATTCAGACAGCGCACTCTGG | YFVprimal_38F | TCCTGGAGGAGCAAAGAAGC | YFVprimal_38R | GGCATCATTGATAGTGCATTGCG |
| YFVprimal_39F | AGGCAATGGATACCATCAGTGTG | YFVprimal_39R | AGATGTGAGTGGGTTTGACGC | YFVprimal_40F | GCATCAGTAGAATGTCTATGGCGA | YFVprimal_40R | TGGTTTTCTCCAGCATGCCTAG |
| YFVprimal_41F | GGCATACCTCATTATTGGCATCCT | YFVprimal_41R | ATTCCAGACAGAGACAGGTTGC | YFVprimal_42F | TGGACAGTGTACGTTGGCATTG | YFVprimal_42R | CCAGGTAAAATGAGAGACCAGTGG |
| YFVprimal_43F | CTGGAATTCAATAACAGTGATGCCTC | YFVprimal_43R | AGAAGCTAGGCTGAGAGCAAGA | YFVprimal_44F | CTCCTGAAATGCCTGCCCTTT | YFVprimal_44R | GATTGTACATGACTCCCACAAAAGC |
| YFVprimal_45F | CACCAGCCTTCTTTGGAATGGA | YFVprimal_45R | AAATGCCTGCGTGCCGTAT | YFVprimal_46F | GGGAACTGAATCTGTTGGACA | YFVprimal_46R | AATCCTTTGACCCCACTCACTTC |
| YFVprimal_47F | TGGAAGGTAGGGTGATTGACCT | YFVprimal_47R | TGTGACCGATGACGATGATGAC | YFVprimal_48F | ATATCCACCGCCTAGAACCAGT | YFVprimal_48R | TGGAATTCCTGGAGAGAGGGTT |
| YFVprimal_49F | TGCCAGATGTTCTCGAGAAACTG | YFVprimal_49R | CTTGTCTGTCTCAACACTGCGT | YFVprimal_50F | AACATCCCGCCTCCTGATGA | YFVprimal_50R | CGCACTTCCTGAGGTTTTTGTG |
| YFVprimal_51F | GGTTTTATGACAATGACAACCCCTAC | YFVprimal_51R | TTTCATGATCTTCCTAGTTCCCGC | YFVprimal_52F | CTTTTGGACAGCAAAGAGTGTTTAAAG | YFVprimal_52R | CCAGAACTTTGGGTCTTGGACA |
| YFVprimal_53F | ATGCAGCCATTGGAGCTTACC | YFVprimal_53R | AATCCCAGGGCCTCAAACTCA | YFVprimal_54F | CAAAGGGAAGCCGTGCCATAT | YFVprimal_54R | CCTGTTCATCATCAAGGTCTGCC |
| YFVprimal_55F | TGGTGGATTCTACGCGGATGA | YFVprimal_55R | CAGAGCATAAGTCACTACCTGCC | YFVprimal_56F | AGGAGGGAAAGCCTACATGGAT | YFVprimal_56R | ACCACACAGTCGTCTCCACT |
| YFVprimal_57F | ATCAGTTCTGACCAGGCTGGAG | YFVprimal_57R | CTGCCATCCTTCAGCTGTAGTT | YFVprimal_58F | GCCATCAAAAGGGTGGAATGATTG | YFVprimal_58R | AACAGCCAATGACAGTAGCCTC |
| YFVprimal_59F | CAGCAAAGCCTATGCCAACATG | YFVprimal_59R | GGTTAGATAAGGGACATCTCTCCA | YFVprimal_60F | CGCACAACATGGTCGATTCATG | YFVprimal_60R | TGTCCAATCAGCGTTCGGATAC |
| YFVprimal_61F | TGGAATGACCAATAGGGCCAC | YFVprimal_61R | CTCCAGCCGTGGTTTATATCCC | YFVprimal_62F | ATAACCGGGATACAAACCACGG | YFVprimal_62R | GGTTTTTCGCAACCTGGAGG |
| YFVprimal_63F | CACGAGTTTTGCCACTGCTAAG | YFVprimal_63R | CTGGAGGAAAAGCAGAGAACCA | YFVprimal_64F | CAAGAACCCAACTGACACTGG | YFVprimal_64R | CTTCCCTCTTTGTGCCACTG |
| YFVprimal_65F | GCACAAAGAGGGAAGCTCAA | YFVprimal_65R | ACTCCTACCAAGATCATGCTCA | YFVprimal_66F | TATCTTGGGTGCAGCGGTG | YFVprimal_66R | TGGGCAAGCTTCTCTCTTCA |
| YFVprimal_67F | TAGGCCAAGGAAAACGCATG | YFVprimal_67R | GAAAAGGCAGCAATCAACGC | YFVprimal_68F | CCAAGAGATCCCGACAATGC | YFVprimal_68R | TAAGTTAGGGTGGCATGGCA |
| YFVprimal_69F | AGTAAATCCTGTGTGCTAATTGAG | YFVprimal_69R | AGGTCCAGGTCTGTTTCCAA | YFVprimal_70F | CCTCCCACCCCAGAGTAAAA | YFVprimal_70R | CATCCAAAGGTCTGCTTATTCTT |
